# Decellularization and Delipidation Protocols of Bovine Bone and Pericardium for Bone Grafting and Guided Bone Regeneration Procedures

**DOI:** 10.1371/journal.pone.0132344

**Published:** 2015-07-20

**Authors:** Chiara Gardin, Sara Ricci, Letizia Ferroni, Riccardo Guazzo, Luca Sbricoli, Giulia De Benedictis, Luca Finotti, Maurizio Isola, Eriberto Bressan, Barbara Zavan

**Affiliations:** 1 Department of Biomedical Sciences, University of Padova, Padova, Italy; 2 Department of Neurosciences, University of Padova, Padova, Italy; 3 Department of Animal Medicine, Productions and Health, University of Padova, Legnaro, Padova, Italy; Texas A&M University Baylor College of Dentistry, UNITED STATES

## Abstract

The combination of bone grafting materials with guided bone regeneration (GBR) membranes seems to provide promising results to restore bone defects in dental clinical practice. In the first part of this work, a novel protocol for decellularization and delipidation of bovine bone, based on multiple steps of thermal shock, washes with detergent and dehydration with alcohol, is described. This protocol is more effective in removal of cellular materials, and shows superior biocompatibility compared to other three methods tested in this study. Furthermore, histological and morphological analyses confirm the maintenance of an intact bone extracellular matrix (ECM). *In vitro* and *in vivo* experiments evidence osteoinductive and osteoconductive properties of the produced scaffold, respectively. In the second part of this study, two methods of bovine pericardium decellularization are compared. The osmotic shock-based protocol gives better results in terms of removal of cell components, biocompatibility, maintenance of native ECM structure, and host tissue reaction, in respect to the freeze/thaw method. Overall, the results of this study demonstrate the characterization of a novel protocol for the decellularization of bovine bone to be used as bone graft, and the acquisition of a method to produce a pericardium membrane suitable for GBR applications.

## Introduction

In the field of oral surgery and dental implantology, bone deficiency represents the main problem that clinicians have to overcome in order to ensure the implant stability and the complete functional restoration. Bone grafting has emerged as a surgical procedure to make up for the bone deficiency [1]. Bone graft not only replaces the missing bone, but also helps regrowth of lost bone by acting as a scaffold for osteoconduction and as a source of osteogenic and osteoinductive molecules for bone formation [2]. Osteoinduction refers to the ability of the scaffold to recruit multipotent mesenchymal stem cells (MSCs) from the surrounding tissue, and to induce their differentiation into bone-forming osteoblasts. Osteoconduction is a characteristic whereby the scaffold acts as a permanent or resorbable matrix that mechanically support the ingrowth of vessels and new bone from the borders of the defect into and onto its surfaces. Osteogenesis is the synthesis of new bone by cells derived from either the graft or the host [3–5]. An ideal bone graft should function through all three mechanisms by providing a substrate that directs three-dimensional (3D) bone growth, recruits and induces differentiation of resident bone-forming cells, and supplies more bone-forming cells to the recipient site. Another fundamental property of an ideal bone graft is osseointegration, which is the ability to bind to the surrounding bone without an intervening layer of fibrous tissue, allowing incorporation of the graft at the host site. Osseointegration is not an isolated phenomenon but depends on previous osteoinduction and osteoconduction [3].

Bone grafts may result from the patient’s bone (autograft), from human donors (allograft) or from animals (xenograft) [6]. Autogenous bone is still considered the “gold standard”, because of its osteoinductive, osteoconductive and osteogenic properties, and absence of immune response. On the contrary, allografts and xenografts have osteoinductive and osteoconductive characteristics but lack the osteogenic properties of autografts [7,8]. Nevertheless, some restrictions in using autograft in clinical practice exist because of donor site morbidity observed with harvesting procedures and the limited amount of bone available [9]. Allograft is a possible alternative to bone autograft with major limitations associated to rejection, transmission of diseases, and cost [10]. With progression in biomaterials technology, the use of xenograft for human tissue reconstruction is increasing [11]. These animal-derived tissues represent an unlimited supply of available material if they could be processed to be safe for transplantation in humans. Indeed, the disadvantage associated to xenografts is that they may trigger unwanted immunological and inflammatory host reactions [12].

To increase efficiency of bone repair, especially in the management of large osseous defects, bone graft areas often require covering with guided bone regeneration (GBR) membranes [13,14]. The underlying concept of GBR was first introduced more than 50 years ago, when cellulose acetate filters were experimentally used for the regeneration of nerves and tendons [15]. Later, a series of animal studies showed that GBR can predictably facilitate bone regeneration in critical-sized osseous defects, as well as healing of bone defects around dental implants [16–18]. The GBR procedure uses membranes that act as physical barriers to epithelial and connective cell invasion from the surrounding soft tissues, thus providing osteogenic cells, which exhibit slower migration rate, better conditions to perform bone regeneration [19]. The use of a barrier membrane is advantageous to facilitate augmentation of alveolar ridge defects, induce bone regeneration, improve bone-grafting results, and treat failing implants. Several barrier membranes have been developed to serve a variety of functions in clinical applications; all of them must satisfy five main design criteria, as described by Scantlebury [20]: biocompatibility, space-making, cell-occlusiveness, tissue integration and clinical manageability.

Although different bone graft materials and barrier membranes have been developed and their use has been extensively investigated, research is ongoing to generate bone grafts and membranes suitable for dental clinical applications. In order to make xenografts an acceptable alternative to autografts and allografts, several processing and storage methods have been studied. Typically, these scaffolds are produced by the process of decellularization of naturally derived tissues. Decellularization has the finality to completely eliminate the cellular component of the native tissue, while maintaining as much of the structure and composition as possible of the original extracellular matrix (ECM) [21]. The ECM has been shown to modulate the behavior of cells that contact the scaffold either by regulating cell migration, influencing tissue-specific phenotypic differentiation, and inducing constructive host tissue remodeling responses. It is likely that the 3D ultrastructure, surface topology, and composition of the ECM all contribute to these effects [22]. Decellularization can be achieved by a variety of agents and techniques. It typically involves exposure to chemical and biologic agents such as detergents and enzymes, and physical forces that unavoidably cause disruption of the associated ECM. Delipidation is another factor to consider, especially for bone grafts [11]. Indeed, the remaining lipids in bone may represent a barrier to cell invasion, negatively influencing its biocompatibility and osseointegration [23]. Moreover, they can induce giant cell reactions which can increase bone resorption and encapsulating fibrosis [24].

The aim of the present work was to develop new methods for decellularization and delipidation of bovine bone and pericardium for the generation of bone substitutes and membranes to be used in bone grafting and GBR procedures, respectively. Histological, morphological, and molecular *in vitro* analyses have been performed in order to test the structural features and the biocompatibility of the produced scaffolds. Additionally, in vivo experiments were carried out to evaluate the biological properties and the host tissue reactions to the implanted bovine biomaterials.

## Materials and Methods

### Source of bovine bone and pericardium

Fresh bone samples were harvested from epiphysis of bovine femur (18–24 month old), immediately after slaughter from a local slaughterhouse (Macello Pubblico Comunale, Udine, Italy). In the laboratory, bone blocks (3 x 3 x 2 cm) were stored at -80°C until use.

Bovine pericardium samples were obtained from the same animals and delivered to the laboratory in cold PBS plus. PBS plus was made of phosphate buffered saline (PBS) (EuroClone, Milan, Italy), containing 1% Penicillin/Streptomycin (P/S) (EuroClone) and 1% Gentamycin (Invitrogen, Paisley, UK). External fat and adherences were removed; then pericardium strips (3 x 1 cm) were obtained, and stored at -80°C until use.

Bone and pericardium samples were either immediately analyzed—hereafter named as native bone sample (NBS) or native pericardium sample (NPS)—or further processed as described below.

### Decellularization protocols of bovine bone

Decellularization of bovine bone blocks started with a wash in PBS plus. Then, four cycles of thermal shock, each comprising a step at 121°C for 20 min, followed by freezing in liquid nitrogen (-196°C) for 16 h, were carried out. During these passages, bone blocks were immersed in bidistilled water (ddH_2_O); the solution was changed at every cycle. Cellular debris was then removed by washing bone blocks in 1% Triton X-100 (Promega, Madison, WI, USA) for 8 h, followed by a second wash in 0.1% Triton X-100 for 16 h. Triton X-100 was dissolved in ddH_2_O. To remove residual detergent, bone samples were washed two times in ddH_2_O for 24 h. All these steps were conducted under continuous shaking at room temperature (RT) with the rotatory shaker VDRL 711 (Asal Srl, Cernusco s/N, Milan, Italy).

At this point, decellularized bone blocks were reduced to granules with dimensions of 0.25–1 mm using Cutting Mill SM 300 (Retsch GmbH, Haan, Germany), then divided into four groups and processed as described in Table 1.

**Table 1:** Description of the processing methods after decellularization of bone samples.

protocol number	sample acronym	steps
B#1	DBS^a^1	Bone granules were washed in 0.5 M sodium bicarbonate (Sigma-Aldrich, St. Louis, MO, USA) in ddH_2_O for 2 days under continuous shaking at RT; the solution was changed twice a day. Residual bicarbonate was removed with two washes of 24 h each in ddH_2_O. Bone granules were dehydrated with a graded ethanol series (50%, 70%, 96%, 100%) for 2 h each in slow agitation at RT. Bone granules were transferred to cell culture dishes and allowed to dry at RT under a sterile laminar flow hood.
B#2	DBS2	Bone granules were washed in 0.5 M sodium bicarbonate (Sigma-Aldrich, St. Louis, MO, USA) in ddH_2_O for 2 days under continuous shaking at RT; the solution was changed twice a day. Residual bicarbonate was removed with two washes of 24 h each in ddH_2_O. Bone granules were freeze-dried with ScanVac Cool Safe freeze-dryer (LaboGene, Lynge, Denmark) for 8h.
B#3	DBS3	Bone granules were dehydrated with a graded ethanol series (50%, 70%, 96%, 100%) for 2 h each in slow agitation at RT. Bone granules were transferred to cell culture dishes and allowed to dry at RT under a sterile laminar flow hood.
B#4	DBS4	Bone granules were freeze-dried with ScanVac Cool Safe freeze-dryer (LaboGene) for 8 h.

^a^Decellularized Bone Sample

### MTT assay

To determine the presence of viable cells in bone samples after decellularization, the MTT based proliferation assay was performed according to the method of Denizot and Lang with minor modifications [25]. Briefly, tissue samples were incubated for 3 h at 37°C in 1 mL of 0.5 mg mL^-1^ MTT solution prepared in PBS. After removal of the MTT solution by pipette, 0.5 mL of 10% DMSO in isopropanol was added to extract the formazan in the samples for 30 min at 37°C. For each sample, optical density (O.D.) values at 570 nm were recorded in duplicate on 200 μL aliquots deposited in microwell plates using a multilabel plate reader (Victor 3, Perkin Elmer, Milano, Italy).

### DNA and RNA content

The DNA content was determined using a DNeasy Blood and Tissue kit (Qiagen) to isolate total DNA from known masses of bone samples following the manufacturer’s protocol for tissue isolation, using overnight incubation in proteinase K (Qiagen).

Total RNA from either native or decellularized tissue samples of known masses was isolated using RNeasy Mini Kit (Qiagen), including DNase digestion with the RNase-Free DNase Set (Qiagen).

The DNA and RNA quality and concentration was measured using the NanoDrop ND-1000 (Thermo Scientific, Waltham, MA, USA) and expressed as nanograms per milligram of dry tissue.

### Lipid content

Oil Red O quantification was used to evaluate the residual lipid content in decellularized bone samples. Oil Red O stock solution was prepared by dissolving 3.5 mg mL^-1^ Oil Red O (Sigma-Aldrich) in isopropanol. Working solution consisted of 3:2 Oil Red O stock in ddH_2_O. About 100 mg of dry bovine bone was incubated in 0.5 mL of Oil Red O working solution for 15 min at RT. After four washing in ddH_2_O, Oil Red O was extracted with 0.25 mL 100% isopropanol. For each sample, O.D. values at 490 nm were recorded using a multilabel plate reader (Victor 3).

### In vitro cytotoxicity test on bovine bone granules

The cytotoxicity of the decellularized bovine bone was evaluated *in vitro* using a mouse-derived established cell line of L929 fibroblasts (Cell bank Interlab Cell Line Collection, Genova, Italy). L929 cells were seeded at a density of 4 × 10^4^/well in 24-well plates for 24 h in cDMEM medium. cDMEM was made of Dulbecco’s Modified Eagle Medium (DMEM) (Lonza S.r.l., Milano, Italy), supplemented with 10% Fetal Bovine Serum (FBS) (Bidachem S.p.A., Milano, Italy) and 1% P/S. Cytotoxicity was assessed with the direct cell contact method, by placing about ten bone granules of the tests materials DBS1, DBS2, DBS3, and DBS4 in each well. The negative control consisted of fibroblasts seeded in presence of a titanium (Ti) disc; blank was obtained seeding fibroblasts in cDMEM with no test material added. Three samples were prepared for each group. The cytotoxicity produced for each different group was assessed with a 48 h cell exposure. After removing the test materials and medium, 1 mL of 0.5 mg mL^-1^ MTT solution was placed in each well. The MTT assay was then performed as explained in the *MTT assay* paragraph.

### Scanning Electron Microscopy (SEM)

For SEM imaging, bone samples were fixed in 2.5% glutaraldehyde in 0.1 M cacodylate buffer for 1 h, progressively dehydrated in ethanol, then critical point-dried followed by gold-palladium coating. The SEM analysis was carried out at the Interdepartmental Service Center C.U.G.A.S. (University of Padova, Italy) by JSM 6490 JEOL SEM.

### Histological analysis

For histological examinations, bone samples were fixed in 4% paraformaldehyde solution in PBS overnight, decalcified with 10% EDTA (Sigma-Aldrich) pH 7.2 for 7 days, then paraffin-embedded and cut into 7 μm thick sections. For Hematoxylin&Eosin (H&E) staining, bone sections were stained with the nuclear dye Hematoxylin (Sigma-Aldrich) and the counterstain Eosin (Sigma-Aldrich).

### Seeding of human Adipose-Derived Stem Cells (ADSCs) onto bovine bone granules

Adipose tissue was digested and the cells isolated and expanded as previously described [26]. At confluence, ADSCs were harvested by trypsin treatment, then cultivated up to passage 3 (p3). Cells at p4 were seeded at density of 1 x 10^6^ cm^-2^ onto DBS3 granules in a 24-well culture plate. The 3D cultures were incubated at 37°C and 5% CO_2_ for up to 28 days in cDMEM, changing the medium every 2 days.

Cell proliferation rate was then evaluated after 3, 7, 14 and 28 days from seeding with the MTT assay. Expression of osteoblast-specific markers in ADSCs cultured 7 and 28 days onto DBS3 granules was measured by Real-time PCR.

### Real time PCR

For the first-strand cDNA synthesis, 1000 ng of total RNA of each sample was reverse transcribed with M-MLV Reverse Transcriptase (Invitrogen, Carlsbad, CA, USA), following the manufacturer’s protocol. Human primers were selected for each target gene with Primer 3 software (S1 Table). Real-time PCRs were carried out using the designed primers at a concentration of 300 nM and FastStart SYBR Green Master (Roche Diagnostics, Mannheim, Germany) on a Rotor-Gene 3000 (Corbett Research, Sydney, Australia). Thermal cycling conditions were as follows: 15 min denaturation at 95°C; followed by 40 cycles of denaturation for 15 sec at 95°C; annealing for 30 sec at 60°C; and elongation for 20 sec at 72°C. Differences in gene expression were evaluated by the 2ΔΔCt method [27]. ADSCs cultured for 7 and 28 days onto tissue culture polystyrene (TCP) in cDMEM were used as control condition. Values were normalized to the expression of the glyceraldehyde-3-phosphate dehydrogenase (GAPDH) internal reference, whose abundance did not change under our experimental conditions.

### Sheep sinus augmentation surgical procedure

The *in vivo* study here described was approved by the Institutional Animal Care and Use Committee of Padova University. Four adult sheep, two years old and 40–50 Kg of weight, were bred according to the European community guidelines (E.D. 2010/63/UE) before performing bilateral sinus augmentation [28]. DBS3 bovine granules were inserted in the inferior osseum septum of the sinuses. Animals were quarantined for 2 weeks to check the general healthy status. Surgical procedures were then carried out in the authorized Veterinary Hospital of Padova University. The animals were euthanized to explant grafted sinuses at 15 and 30 days post intervention (p.i.). For histological analyses, bone grafts were processed as described in the *Histological analysis* paragraph and stained with H&E.

### Decellularization protocols of bovine pericardium

Bovine pericardium was decellularized using two protocols based on either osmotic shock or freeze/thaw associated with enzymes. These passages are detailed in Table 2. Bovine pericardium strips obtained from both protocols were then subjected to a decontamination step, consisting in an incubation with 30% isopropanol for 24 h, changing the solution after 8 h. Pericardium samples were then washed twice with PBS for 24 h at RT, and stored in PBS at +4°C until being analyzed.

**Table 2:** Description of the processing methods of pericardium decellularization.

*protocol number*	*sample acronym*	*steps*
P#1	DPS^a^1	Strips of bovine pericardium were treated with an hypotonic buffer (ten times diluted PBS) containing 10% dimethyl sulfoxide (DMSO) (Sigma-Aldrich) and 10 mM ascorbic acid (Sigma-Aldrich), in presence of protease inhibitors (PI) (Roche, Basel, Switzerland) for the first 8 h, and without PI for the subsequent 16 h; all these steps were conducted at 4°C with agitation. Pericardium samples were washed in 1% Triton X-100 for 8 h, then in 0.1% Triton X-100 for 16 h at 4°C with agitation. Triton X-100 was prepared in hypotonic buffer. Strips of bovine pericardium were then treated with an hypertonic solution made of 0.5 M sodium chloride (Sigma-Aldrich) in PBS for 24 h at RT with agitation; the solution was changed twice. Pericardium samples were washed two times with 10 mM sodium deoxycholate (SD) (Sigma-Aldrich) dissolved in hypotonic buffer for 24 h at RT with agitation. Residual detergent was removed with two washes of 24 h each in hypotonic buffer.
P#2	DPS2	Strips of bovine pericardium were exposed to two cycles of dry freeze/thaw, followed by two cycles of freeze/thaw in hypotonic solution; in both cases, freezing was at -80°C for 2 h, then samples were left to thaw on the bench for 2–4 h. Pericardium samples were then treated with hypotonic buffer containing 0.1% SD, 0.1% ethylenediaminetetraacetic acid (EDTA) (Sigma-Aldrich), in presence of PI for 8 h at RT under continuous shaking. This was followed by a wash in hypotonic buffer for 16 h. Bovine pericardium strips were incubated in an enzymatic solution made of 50 U mL^-1^ DNase (Qiagen GmbH, Hilden, Germany) and 1 U/mL RNase (Sigma-Aldrich) in PBS containing 10 mM magnesium chloride (Sigma-Aldrich) and 50 ug/mL bovine serum albumin (BSA) (Sigma-Aldrich) for 3 h at 37°C. Pericardium samples were washed twice with PBS for 24 h at RT under slow agitation.

^a^Decellularized Pericardium Sample

### MTT assay, DNA and RNA content

To determine the presence of viable cells in pericardium samples after decellularization, the MTT assay was performed as described for bone in the *MTT assay* paragraph.

DNA and RNA were isolated from pericardium samples as reported above in the *DNA and RNA content* paragraph. The only difference was that the nucleic acids concentration was expressed as micrograms per milligram of dry tissue.

### Seeding of human fibroblasts onto bovine pericardium

Before seeding the cells, squares (1 x 1 cm) of decellularized pericardium samples were cut and laid down on a 24-well culture plate using sterile forceps and scissors. Human fibroblasts were then seeded at a density of 1 x 10^6^ cm^-2^ in presence of cDMEM for 7 days.

### SEM and Histological analyses

For SEM imaging, pericardium samples were processed and analyzed as described for bone in the *Scanning Electron Microscopy (SEM)* paragraph.

For histological examinations, formalin-fixed and paraffin-embedded pericardium samples were cut into 7 μm thick sections. Pericardium sections were then stained with H&E, or with Weigert’s solution (Sigma-Aldrich) in order to visualize elastic fibers.

### Rat subcutaneous implantation

The protocol for the *in vivo* study was performed as approved by the Institutional Animal Care and Use Committee of Padova University. Decellularized pericardium samples were implanted in the abdominal area of three adult female Lewis rats (Charles River Laboratories), weighing 150–200 g, as described by Silva-Correia et al. [29]. After 7 days of implantation, the animals were sacrificed by an overdose of gaseous anesthetic, and the implants with surrounding subcutaneous tissue were retrieved for histological examinations after H&E staining.

### Statistical analysis

Results are reported as mean ± standard deviation. One-way analysis of variance (ANOVA) Bonferroni’s post hoc test was applied to identify any significant changes between groups in the MTT, DNA/RNA, Oil Red O, and real time PCR data. Differences were considered statistically significant at P<0.05Statistical analyses were performed using the SPSS 16.0 software package (SPSS Inc., Chicago, IL, USA).

## Results and Discussion

### Decellularization protocols of bovine bone

The use of bone grafting materials associated with the GBR technique seems to provide the most promising result to restore bone defects in dental clinical practice. A variety of decellularized tissues have been used as scaffold for bone regeneration applications [11–14].

In the first part of our work, we compared four different methods of bovine bone decellularization and delipidation. Epiphysis of bovine femur was chosen as source of starting material, due to its composition. Epiphysis is made almost entirely of spongy cancellous bone, which is considered more osteogenic than cortical bone [9]. Indeed, the presence of spaces between the structure of cancellous bone, and the large surface area, makes it very attractive at sites where new bone formation is desired. Although cancellous bone lacks mechanical strength, it is a good space filler and mostly preferred for repairing dental defects [2].

The optimal decellularization strategy would involve the use of the mildest protocol possible that yields an acellular material without disruption of the structural and functional component of the ECM. The physical and chemical methods described in this study have been already applied to a variety of tissues and organs, as widely reported in the literature [21,22,30]. However, the specific combination of these treatments and the duration of each step, as well as the proper concentration of the reagents used, have been conceived, designed and developed in our work. All the bone decellularization protocols started with four cycles of thermal shock (from -196°C to 121°C) in ddH_2_O. This step, generally used at the beginning of the decellularization protocol, is useful for disrupting cellular membranes and causing cell lysis [30]. Cellular debris was then removed from all the bone samples washing with Triton X-100. Triton X-100 is a non-ionic detergent, that can disrupt lipid-lipid and lipid-protein interactions, leaving protein-protein interactions intact [31]. This passage was conducted under mechanical agitation in order to increase the effectiveness of the detergent diffusion through the bone. Decellularized bone samples were then reduced to granules of dimensions ranging from 0.25 to 1 mm. It is well accepted that the particle size may interfere with the success of the regenerative therapy [32]. In particular, it has been reported that particle sizes ranging from 0.125 to 1 mm possess a higher osteoinductive effect than do particles below 0.125 mm [33]. After grinding, bovine bone granules were divided into four groups and subjected to different additional steps: washing with sodium bicarbonate and dehydration with graded ethanol for DBS1; washing with sodium bicarbonate and lyophilization for DBS2; dehydration with graded ethanol for DBS3; lyophilization for DBS4. The sterility of the produced materials was achieved by exposing bone samples to a 25 kGy dose of gamma irradiation. In order to identify the best decellularization protocol, we evaluated their efficiency in terms of removal of all cellular materials, including nucleic acids and lipids, as well as their in vitro cytotoxicity.

### Evaluation of cell removal, residual nucleic acids and lipid content in decellularized bovine bone granules

**Fig 1:**
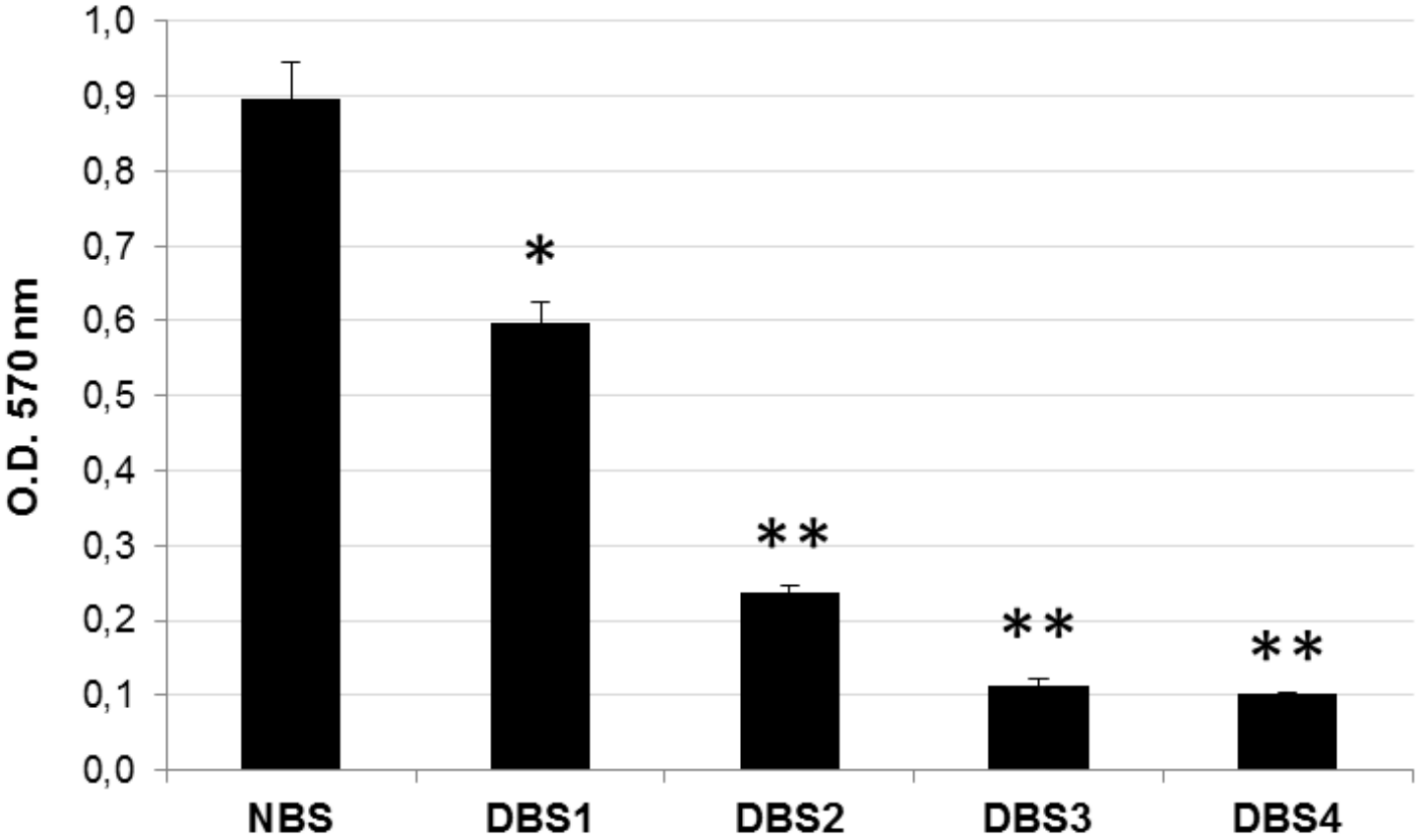
MTT assay on decellularized bovine bone. Quantification analysis of amount of residual cells in decellularized bone samples DBS1, DBS2, DBS3, and DBS4 compared to NBS, estimated with MTT assay. Values are expressed as mean ± standard deviation (n = 3 per group). Statistically significant differences are indicated as *P<0.05 and **P<0.01 and compared with NBS.

The MTT assay was performed for assessing the residual cell vitality in the four decellularized bone samples. Native bone (NBS) was used as positive control. As shown in Fig 1, protocols B#3 (DBS3) and B#4 (DBS4) resulted in a significant (P<0.01) removal of cells when compared to the first two decellularization methods. The additional washing step with sodium bicarbonate may explain the slightly worst results obtained with protocols B#1 and B#2 (P<0.05 and P<0.01, respectively).

**Fig 2:**
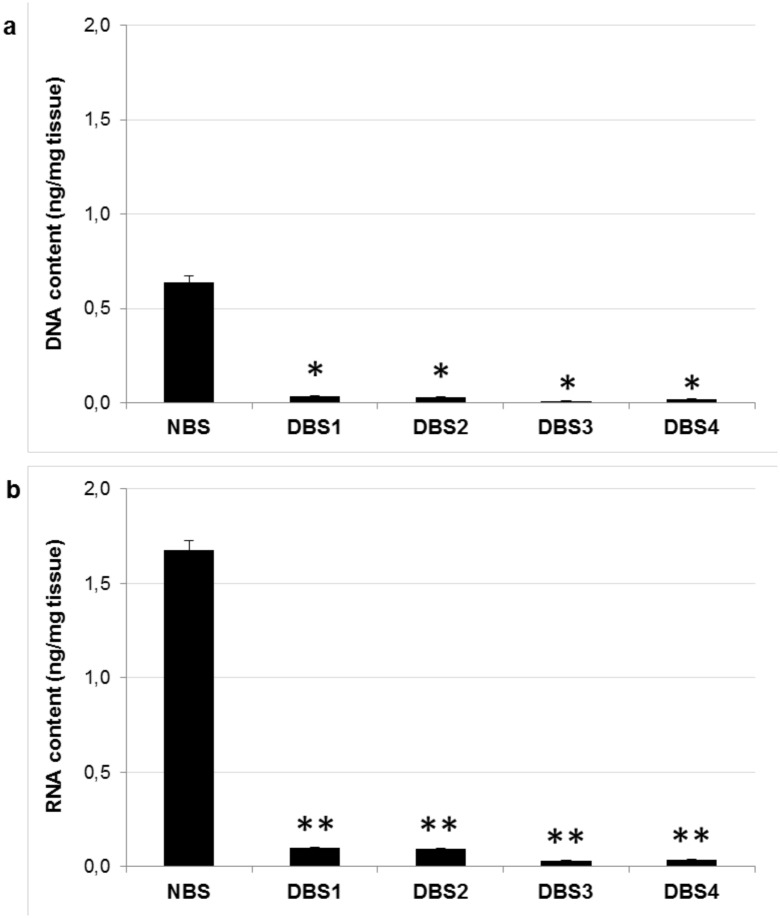
Nucleic acids quantification in decellularized bovine bone. Quantification analysis of amount of residual (a) DNA and (b) RNA in decellularized bone samples DBS1, DBS2, DBS3, and DBS4 compared to that of NBS. Content of residual DNA and RNA was normalized by dry weight of each specimen. Values are expressed as mean ± standard deviation (n = 3 per group). Statistically significant differences are indicated as *P<0.05 and **P<0.01 and compared with NBS.

The quantification of nucleic acids in all the four samples is shown in Fig 2. In detail, the average DNA content was: 0.037 ± 0.005 ng/mg for DBS1; 0.031 ± 0.006 ng/mg for DBS2; 0.011 ± 0.003 ng/mg for DBS3; 0.019 ± 0.002 ng/mg for DBS4. The average RNA content was: 0.098 ± 0.004 ng/mg for DBS1; 0.093 ± 0.007 ng/mg for DBS2; 0.029 ± 0.003 ng/mg for DBS3; 0.036 ± 0.004 ng/mg for DBS4. The reduction in both DNA and RNA content was significant (P<0.05 and P<0.01, respectively) and higher than 90% compared to that of native bone (0.639 ± 0.048 ng DNA/mg; 1.678 ± 0.058 ng RNA/mg), indicating that cells and their nuclear materials have been effectively removed. This result is particularly important because it would ensure that the bone tissue is essentially devoid of immunogenic active molecules [33].

**Fig 3:**
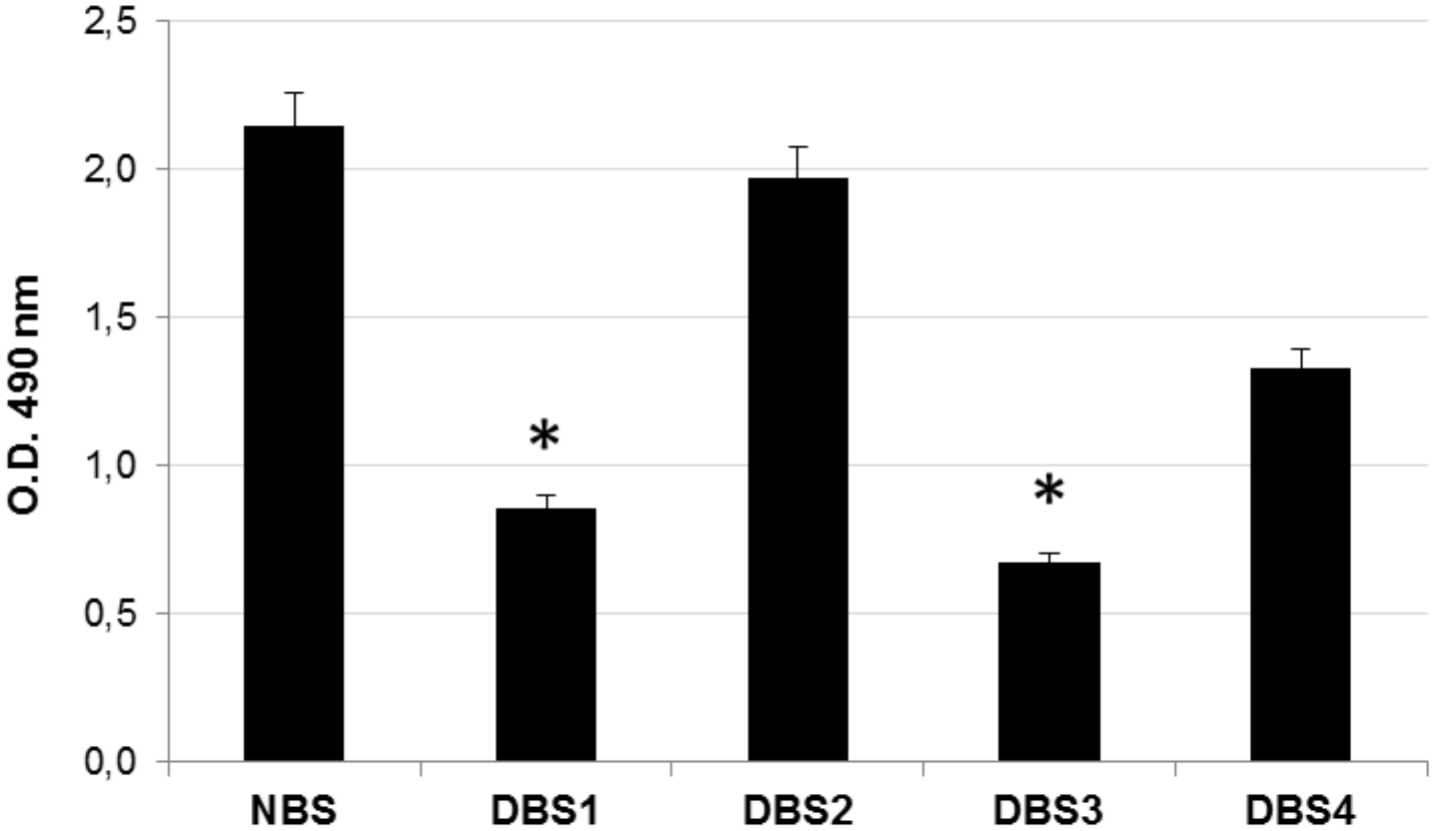
Lipids quantification in decellularized bovine bone. Quantification analysis of residual lipid content in bone samples DBS1, DBS2, DBS3, and DBS4 compared to NBS, estimated with Oil red O quantification. Values are expressed as mean ± standard deviation (n = 3 per group). Statistically significant differences are indicated as *P<0.05 and compared with NBS.

Oil red O staining was then used to evaluate the residual lipid content in bone samples. As an azo dye, Oil Red O can combine with triacylglycerol to give out jacinth lipid droplets, which can be spectrophotometrically quantified [34]. As evident from Fig 3, protocols B#1 and B#3 produced a significant (P<0.05) removal of lipids from bone granules. On the contrary, bovine bone samples processed with protocols B#2 and B#4 did not achieve an appropriate lipid elimination. It is reasonable to think that treatment with ethanol positively contributed to removal of lipids from bone. This result should be taken strongly into account, since delipidation is an important procedure for the preparation of bone grafts because residual lipids negatively affect the osseointegration [11].

### Evaluation of cytotoxicity of decellularized bovine bone granules

**Fig 4:**
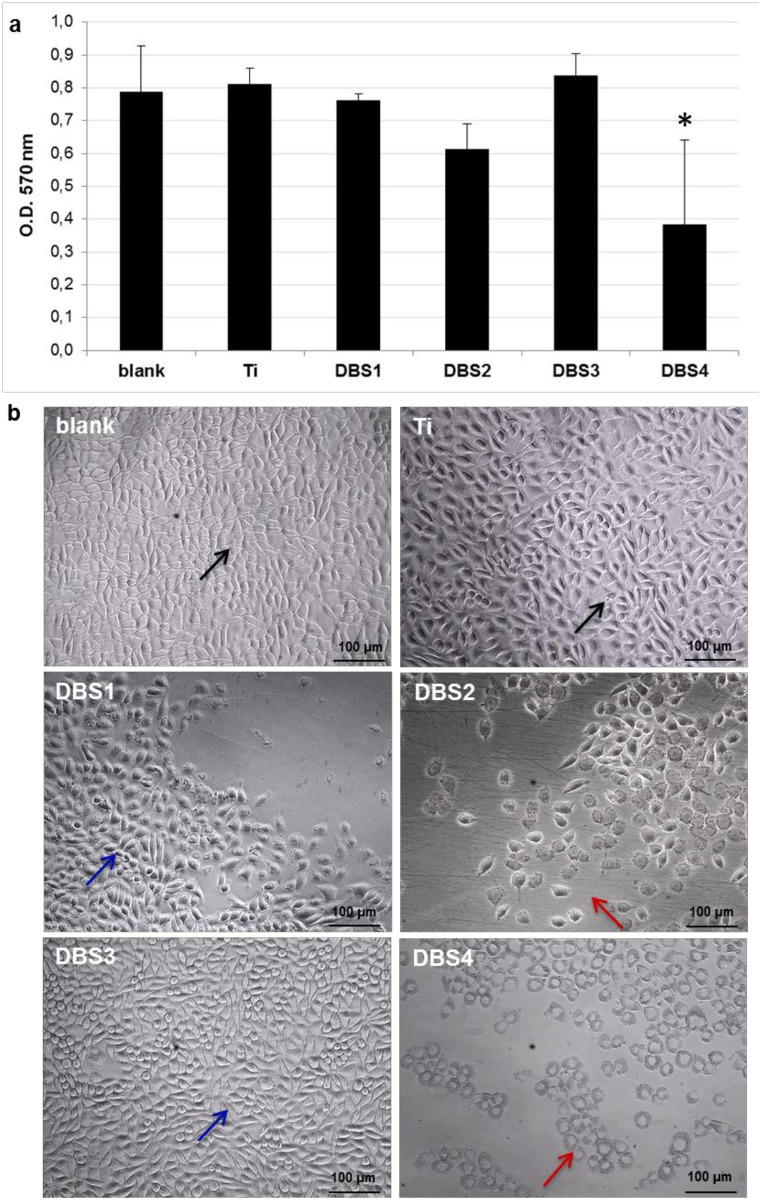
In vitro cytotoxicity test on decellularized bovine bone granules. (a) O.D. values at 570 nm representing proliferation rates of L929 fibroblasts cultured in direct contact with DBS1, DBS2, DBS3, DBS4 granules, titanium (Ti) disc, and without granules (blank) (n = 3 per group) Statistically significant differences are indicated as *P<0.05 and compared with blank. (b) Morphology of L929 fibroblasts cultured in presence of the test materials cited above. Images were acquired after removal of the bone granules. Cells (blue arrows) grown in contact with DBS1 and DBS3 granules show a morphology similar to that of cells (black arrows) contacting the Ti disc or blank. The morphology of cells (red arrows) in contact with DBS2 and DBS4 granules is altered.

When a material has to be used in living tissues, excellent biocompatibility is essential in order to avoid any adverse effect [35]. *In vitro* cytotoxicity represents an initial and critical step to evaluate the biocompatibility of the ideal material. The cytotoxicity of bone samples produced in this work was quantitatively measured with MTT assay, where a higher cell proliferation rate is expected for biocompatible candidate. The cytotoxicity was estimated by directly exposing L929 fibroblasts to DBS1, DBS2, DBS3 and DBS4 granules. Cells cultivated in absence of bone granules or with a Ti disc were used as blank and negative control condition, respectively. The MTT results reported in Fig 4a show that L929 cells grew well in presence of DBS3 or DBS1 granules, with absorbance values quite similar to those of blank and negative control. On the contrary, cell proliferation rate was negatively affected by the presence of DBS2 and DBS4 (P<0.05) granules.

In accordance with the MTT results, L929 cells were visualized to grow directly underneath DBS3 granules without necrosis or detachment, presenting a morphology similar to both negative control and blank samples (Fig 4b). On the contrary, the cell morphology was compromised for L929 cells cultured with DBS2 and DBS4 granules, indicating the inhibition of cell growth and a certain degree of cytotoxicity. It is likely that treatment with ethanol provided for DBS1 and DBS3 promoted the total removal of cellular debris, conferring them superior biocompatibility.

### Morphological and histological analyses of decellularized bovine bone

**Fig 5:**
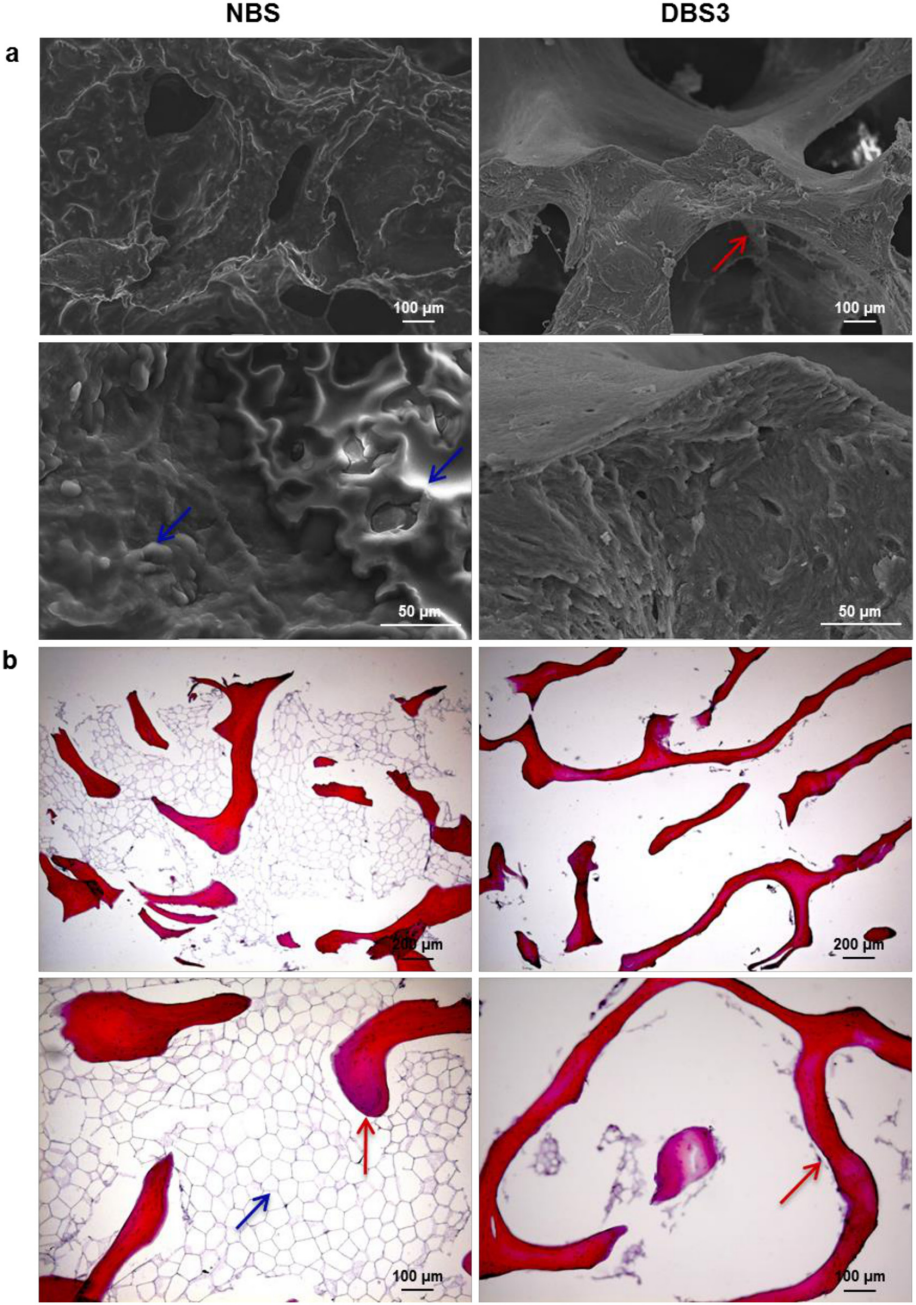
Morphological analyses of bovine bone. (a) Representative SEM micrographs of native (NBS) and decellularized (DBS3) bone samples. In DBS3, the typical structure of cancellous bone with bone trabeculae (red arrow) is well evident; whereas it is hidden by a layer of fatty material (blue arrows) in NBS. (b) H&E staining of NBS and DBS3. Adipose tissue (blue arrow) between bone trabeculae (red arrows) is visible in NBS but it has been completely removed after decellularization in DBS3.

In the light of the results presented so far, in our opinion bovine bone samples decellularized with protocol B#3 gave better results compared to the other protocols in terms of native cells elimination, nucleic acids and lipid removal, and lack of cytotoxicity. For this reason, further analyses were conducted exclusively on DBS3. SEM images demonstrate that the morphology of DBS3 is not altered by the decellularization protocol. Indeed, the typical structure of cancellous bone with bone trabeculae is well preserved, whereas the lipid component has been completely removed (Fig 5a). This is in contrast with the morphology of NBS, whose structure is hidden by a layer of fatty material, which makes its surface smoother. These observations are confirmed by histological analyses (Fig 5b). H&E staining of NBS clearly shows the presence of fat cells between bone trabeculae. Conversely, the lipid component is absent in the decellularized bone sample, but retains an intact ECM. These results seem to confirm what previously suggested, namely that the final step in graded ethanol, in addition to dry bone granules, strongly contributed to the elimination of any contaminating material. Such a result is very encouraging since one of the fundamental challenges of this work was to find an effective method to defat the produced bone granules. Different chemical or physical treatments are generally used to carry out this step. Chemical organic solvents, such as chloroform, dichloromethane or acetone, may leave toxic residues in the grafts as well as being harmful to the health of operators. On the other hand, physical methods of lipid removal such as supercritical CO_2_, although efficient in defatting bone samples, need expensive equipment and solvents [36]. Recently, Zhang and co-workers demonstrated that the use of lipase could eliminate fat from porcine bone similarly to acetone, but in a shorter time and without toxic effects [11]. Although lipase is extensively used in various applications in many fields, its activity is strongly pH and temperature dependent, and thus more difficult to control. Furthermore, the influence of treatment with lipase in bone formation has not yet been investigated in vivo. The results reported in this study would indicate that the use of graded ethanol could be strongly considered as an alternative treatment for removing lipids from bone samples.

### Evaluation of the osteoinductivity of decellularized bovine bone in vitro

**Fig 6:**
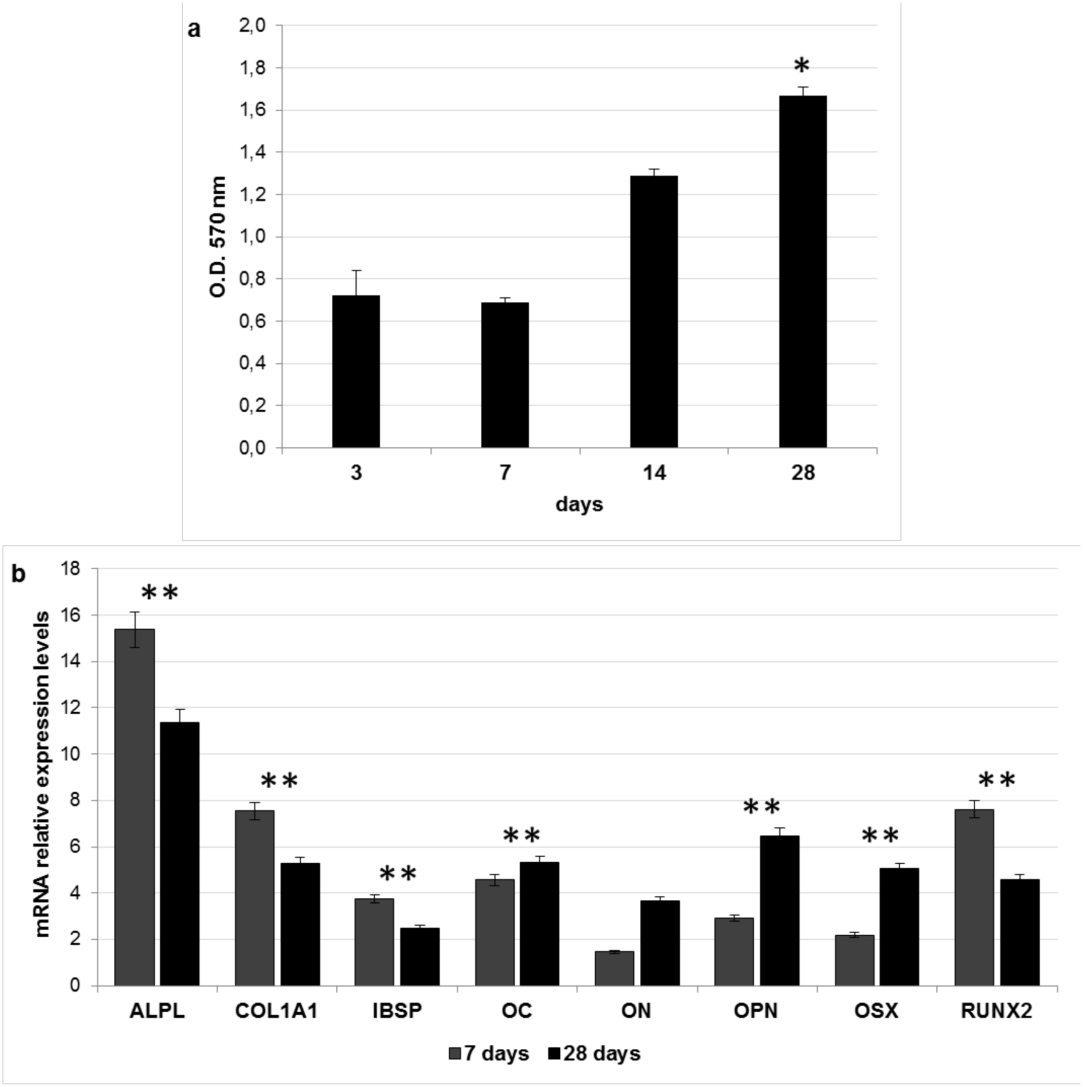
Biological responses of ADSCs seeded onto DBS3 granules. (a) Proliferation rate of ADSCs seeded onto DBS3 granules for 3, 7, 14 and 28 days calculated with MTT assay. Statistically significant differences are indicated as *P<0.05 and compared with the first time point. (b) Expression of osteoblast specific markers after in vitro ADSCs seeding onto DBS3 granules for 7 and 28 days in basal medium. The results are reported as ratio with respect to the mRNA expression of ADSCs seeded on TCP. Statistically significant differences are indicated as **P<0.01 and compared with the control condition.

As described in the Introduction, a key property of an ideal bone graft is its osteoinductivity [2,4,5]. The osteoinductivity of DBS3 was assessed evaluating the ability of ADSCs seeded onto these granules to express osteogenic markers in absence of osteogenic differentiation factors. A preliminary MTT assay indicated that DBS3 granules are able to support the growth of ADSCs, whose proliferation rate increases during the culturing time, with a maximum value at 28 days (P<0.05) (Fig 6a). When ADSCs are cultured on the DBS3 granules, the gene expression of alkaline phosphatase liver/bone/kidney (ALPL), collagen type I alpha 1 (COL1A1), integrin-binding sialoprotein (IBSP), and runt-related transcription factor 2 (RUNX2) is found to be significantly (P<0.01) upregulated after 7 days from seeding, then slightly decreases at 28 days (Fig 6b). ALPL is a marker of early osteogenic development, and has probably an initiator and regulator role in calcification [37]. The elevated ALPL expression observed in this work supports the success of the osteogenic differentiation of ADSCs and might be an indication of the osteoinductive properties of the bone granules used. RUNX2 is one of the transcription factors required for the differentiation of MSCs into preosteoblasts [38]. Several studies report that RUNX2 controls the expression of several bone ECM protein genes, including COL1A1, IBSP, osteocalcin (OC), and osteopontin (OPN), through binding to their promoters [39,40]. The high expression found for COL1A1 at 7 days is very interesting since collagen synthesis is known to be a prerequisite for ECM formation and mineralization in bone [41]. IBSP and OPN are ECM glycoproteins implicated in the regulation of mineralized nodule nucleation [42]. The results presented in this study might confirm the role of RUNX2 in stimulating the expression of both IBSP and OPN [43]. RUNX2 is essential for the commitment of MSCs to the osteoblast lineage, but its expression has to be downregulated for bone maturation [44]. Osterix (OSX) is considered a downstream gene of RUNX2, and plays a fundamental role in the commitment of preosteoblastic cell differentiation into mature osteoblasts [45]. In our long-term cultures, OSX expression becomes predominant on that of RUNX2 (P<0.01). The acquisition of a mature osteoblast phenotype could also be explained by the significant (P<0.01) increase in the expression of OC over time. OC is the most abundant non-collagenous protein in bone ECM after collagens and a marker of mature osteoblasts [46]. Finally, osteonectin (ON) is a glycoprotein that binds calcium [47]. It is secreted by osteoblasts during bone formation, initiating mineralization and promoting mineral crystal deposition. ON also shows affinity for collagen in addition to bone mineral calcium. In this study, the expression levels of ON increases at 28 days of culture. Taken together, our results seem to demonstrate that the upregulation of several osteogenic genes in ADSCs may be dependent on the DBS3 granules surface characteristics.

### Evaluation of the osteoconductivity of decellularized bovine bone in vivo

**Fig 7:**
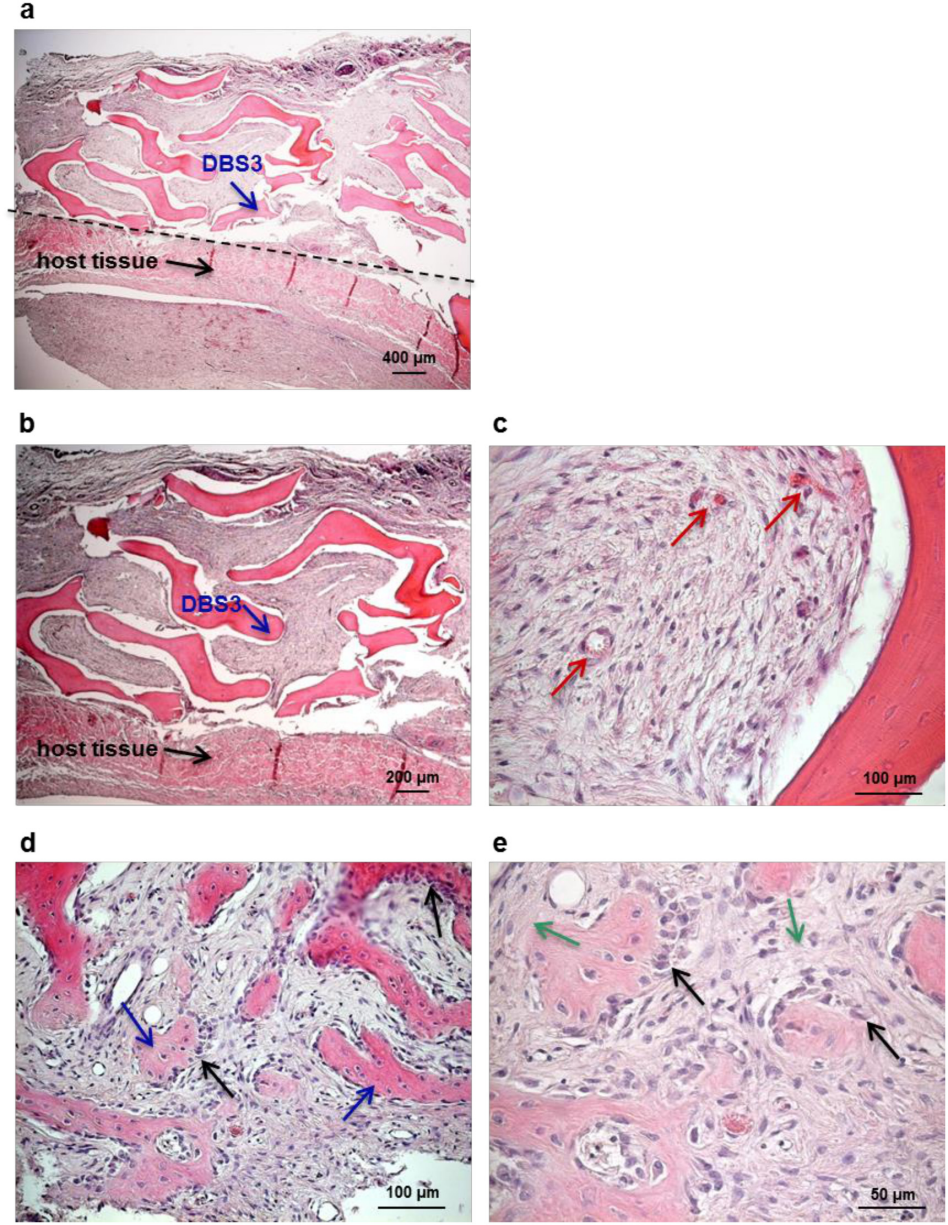
Morphological analysis of sinus explants 15 days p.i.. (a) Histological overview of the sinus area showing a scarce integration of the biomaterial (DBS3 granules, blue arrow) within the host bone tissue (black arrow). (b) Higher magnification of the implantation area, displaying scarce contact between DBS3 granules (blue arrow) and host bone tissue (black arrow). (c) Presence of blood vessels (red arrows) in the augmentation area. (d) Ovine osteoblats (black arrows) adhering to DBS3 granules (blue arrows). (e) Higher magnification of osteoblasts (black arrows) producing new bone ECM (green arrows).

**Fig 8:**
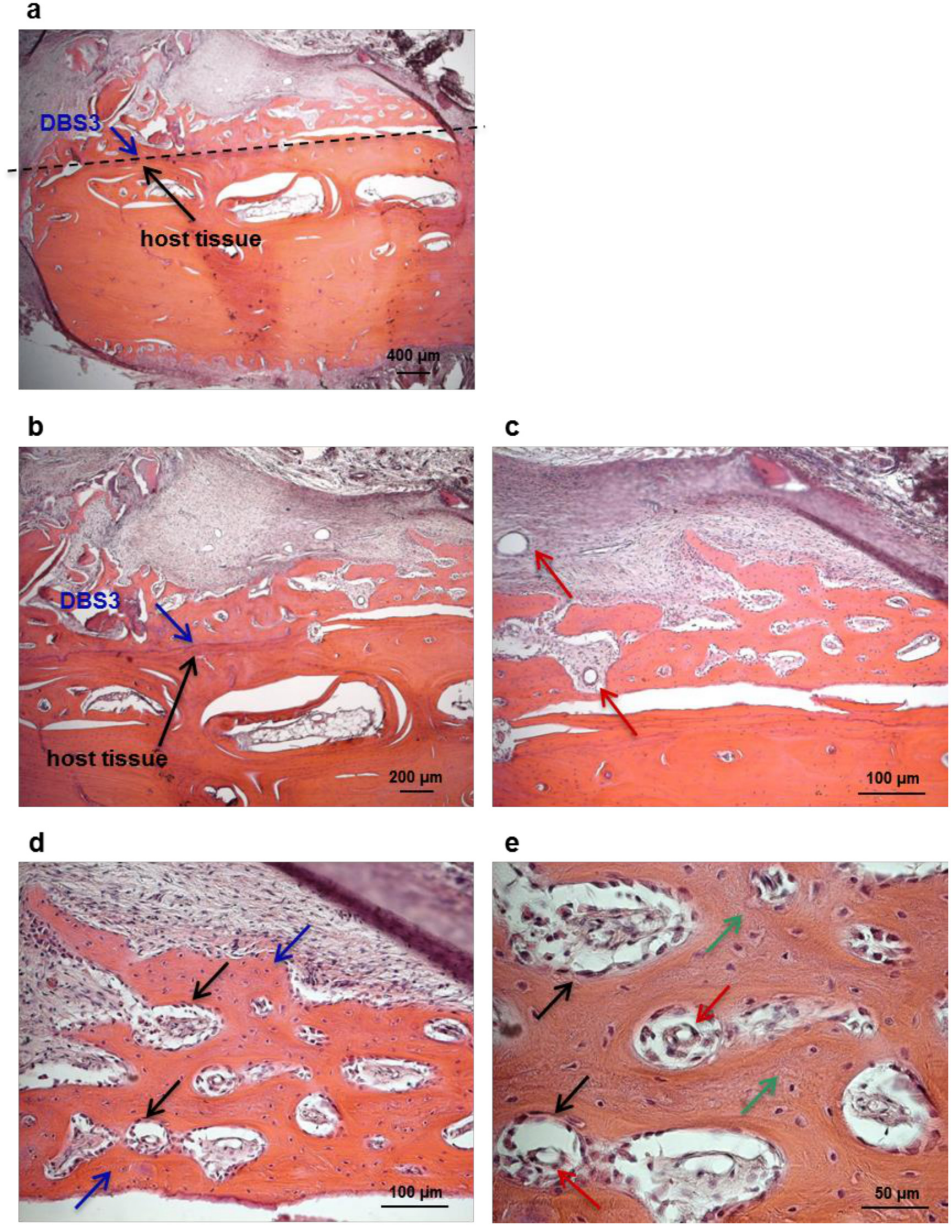
Morphological analysis of sinus explants 30 days p. i.. (a) Histological overview of the sinus area, showing the direct contact between the biomaterial (blue arrow) and host bone (black arrow). (b) Higher magnification of the implantation area, demonstrating that DBS3 granules (blue arrow) are well integrated within the host bone tissue (black arrow). (c) Presence of blood vessels (red arrows) in the augmentation area. (d) Ovine osteoblasts (black arrows) have colonized the bovine granules (blue arrows) and started to lay down new bone ECM. (e) Higher magnification of the new bone ECM (green arrows) synthesized by ovine osteoblasts (black arrows) containing blood vessels (red arrows).

An essential goal for the development of a bone graft material to be used in a clinical setting is osteoconductivity [2,4,5]. The osteoconductivity of DBS3 granules was evaluated in a maxillary sinus augmentation model in sheep. Previous investigations have demonstrated that such a procedure in sheep is a reliable model to evaluate bone formation in humans [48,49]. The surgical procedure was uneventful for all animals. All sheeps did not show any p.i. complications nor clinical symptoms of maxillary sinusitis. Sinus explants evaluated macroscopically 15 and 30 days after augmentation reveal that DBS3 bovine granules are inserted in the surrounding host tissue, where no signs of inflammation or adverse tissue reactions are present. From an histological point of view, the interaction between the biomaterial particles and the host bone tissue is limited at 15 days (Fig 7a and 7b); however, their integration in the augmentation area increases over time (Fig 8a and 8b). This is also demonstrated by the presence of ovine connective tissue that completely surrounds the implanted biomaterial. The presence of blood vessels is already detectable in sinus explants at 15 days (Fig 7c), and they become more defined after 30 days from biomaterial implantation (Fig 8c). This result is very encouraging since vascularization is a prerequisite for the production of new bone tissue [4,50]. In the process of new bone formation, osteoblasts play a pivotal role since they are the cells responsible for new ECM deposition. Ovine osteoblasts start to colonize the implanted DBS3 granules at 15 days (Fig 7); the adhesion of osteoblasts to the biomaterial is clearly visible at a higher magnification (Fig 7). The biomaterial appears fully infiltrated by these cells after an implantation period of 30 days (Fig 8d). At this stage, the presence of newly synthesized bone is significant. This new woven bone appears as vital bone tissue, containing osteocytes inside bone lacunae, and blood vessels in between the bone voids (Fig 8e). Based on the histological results of this study, we can conclude that the DBS3 bovine granules determine a substantial amount of new bone formation and a proper blood supply, thus representing a suitable bone graft candidate material.

### Decellularization protocols of bovine pericardium

The use of bone grafting material alone seems to be less effective for an optimal bone regeneration than the combination of a supporting material and a barrier [51]. As explained in the Introduction, the GBR technique relies on the presence of a membrane that acts as a barrier for non-osteogenic cell infiltration. The choice of a barrier membrane is a critical step in GBR procedures. Resorbable membranes are generally preferred to non-resorbable membranes because they avoid the need for membrane removal, they have greater cost-effectiveness and decreased patient morbidity [52]. Currently used resorbable membranes are polymeric or collagen derived from different animal sources. Collagen membranes derived from bovine pericardium were shown to be efficient for GBR in rabbit mandibular defects [53]. The structure of pericardium, consisting of a network of collagen and elastic fibers embedded in an amorphous matrix, is unique, and this results not only in a smooth yet porous surface for cellular attachment and proliferation, but also in sufficient density for soft tissue exclusion [54]. In order to generate a resorbable membrane to be used in the GBR technique, in the second part of this study we developed a method of bovine pericardium decellularization. Most of the methods described in the literature for decellularization of bovine pericardium are based on treatments with hypotonic buffer, sodium dodecyl sulfate (SDS), and nuclease solution; or alkaline treatment followed by phosphoric acid washing; or enzymatic digestion [55–57]. In our work, two different protocols were tested and compared: the first (protocol P#1) was based on osmotic shock associated with detergents, the second one (protocol P#2) implied multiple steps of freeze/thaw followed by use of enzymes. The single physical, chemical or enzymatic methods here proposed have been already described previously [21,22,30]. Nevertheless, the appropriate combination and the duration of each treatment, as well as the time of exposure to the various reagents, have been developed and optimized in this study. Osmotic shock was obtained by placing bovine pericardium strips in hypotonic and hypertonic solutions, alternated by washing in non-ionic (Triton X-100) and ionic (SD) detergents. It has been reported that multiple steps in hypotonic/hypertonic solutions achieve the maximum osmotic effect [22]. The hypotonic solution was enriched with PI in order to prevent degradation by enzymes released from disrupted cell compartments. The use of Triton X-100 and SD were then preferred over other detergents in the decellularization process of pericardium because they did not affect the structural integrity of either collagen and elastin, as previously reported [58]. The second decellularization protocol was based on the method described by Stapleton et al. [59] for porcine meniscus with some modifications. Briefly, bovine pericardium samples were exposed to two cycles of dry freeze/thaw, followed by two cycles of freeze/thaw in hypotonic solution. The freeze/thaw technique was adopted to allow ice crystal formation, potentially opening up the membrane to facilitate diffusion of the subsequent solutions. Bovine pericardium strips were then incubated in SD and hypotonic buffer, followed by a treatment with nucleases (DNase and RNase) in order to digest residual nucleic acids. Following decellularization with the two protocols, DPS1 and DPS2 were disinfected with isopropanol. Sterilization was achieved by exposing pericardia to a 25 kGy dose of gamma irradiation.

### Evaluation of cell removal and residual nucleic acids content in decellularized bovine pericardium

**Fig 9:**
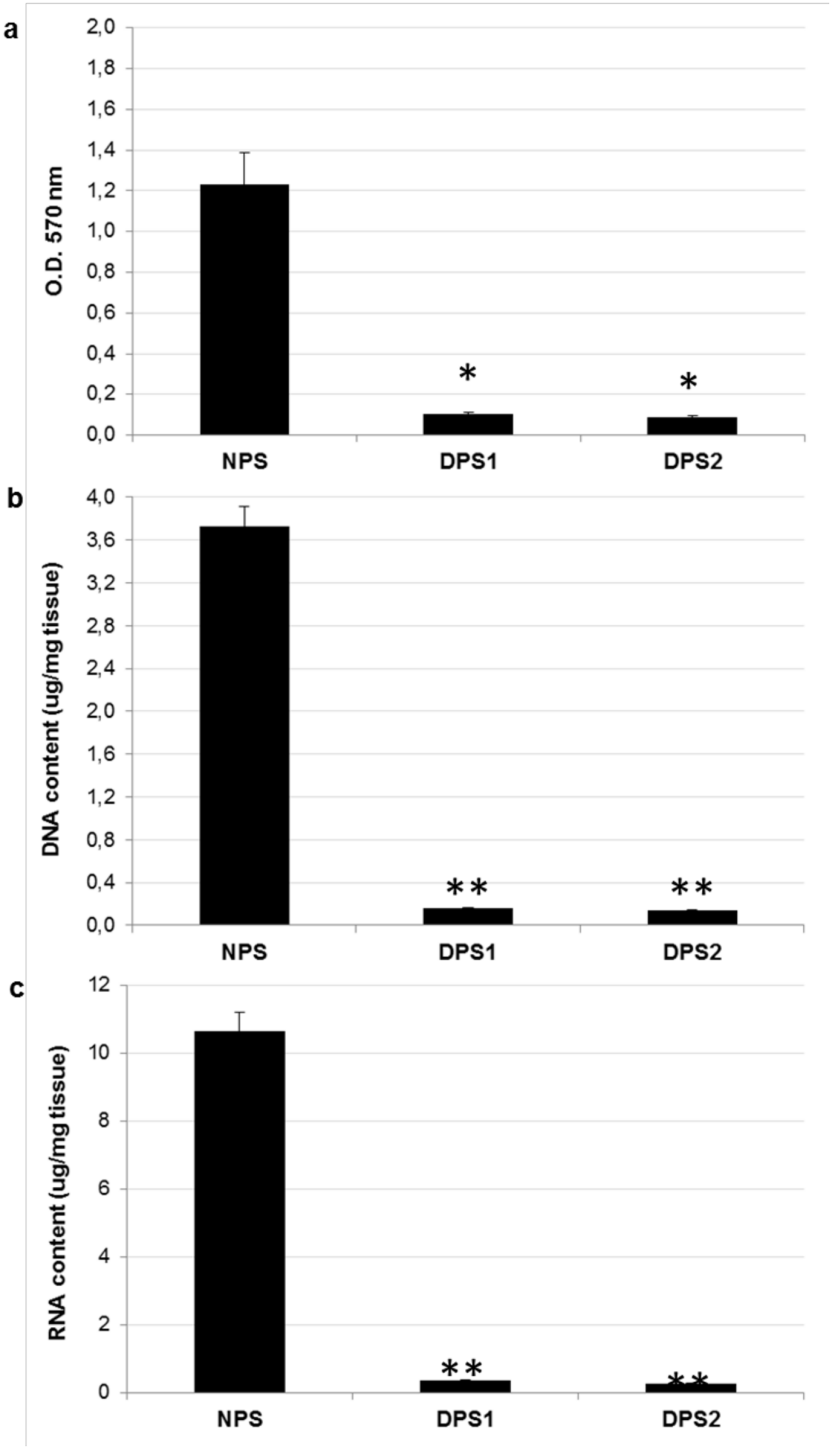
MTT assay and nucleic acids content in decellularized bovine pericardium. Quantification analyses of (a) residual cells, (b) DNA content, and (c) RNA content in pericardium samples DPS1 and DPS2 compared to NPS. Values are expressed as mean ± standard deviation (n = 3 per group). Statistically significant differences are indicated as *P<0.05 **P<0.01 and compared with NPS.

At this point, the efficiency of decellularization protocols in terms of cell removal, and reduction of DNA and RNA content, was analyzed and compared. Both protocols produced an equivalent and good (P<0.05) removal of native cells (Fig 9a), and a reduction in the content of DNA (Fig 9b) and RNA (Fig 9c) higher than 90% when compared to that of the native pericardium (NPS) (P<0.01). In detail, the average DNA content was: 3.730 ± 0.178 μg/mg for NPS; 0.160 ± 0.006 μg/mg for DPS1; 0.142 ± 0.007 μg/mg for DPS2. The average RNA content was: 10.676 ± 0.434 μg/mg for NPS; 0.368 ± 0.016 μg/mg for DPS1; 0.276 ± 0.012 ng/mg for DPS2.

### Biological properties of decellularized bovine pericardium

**Fig 10:**
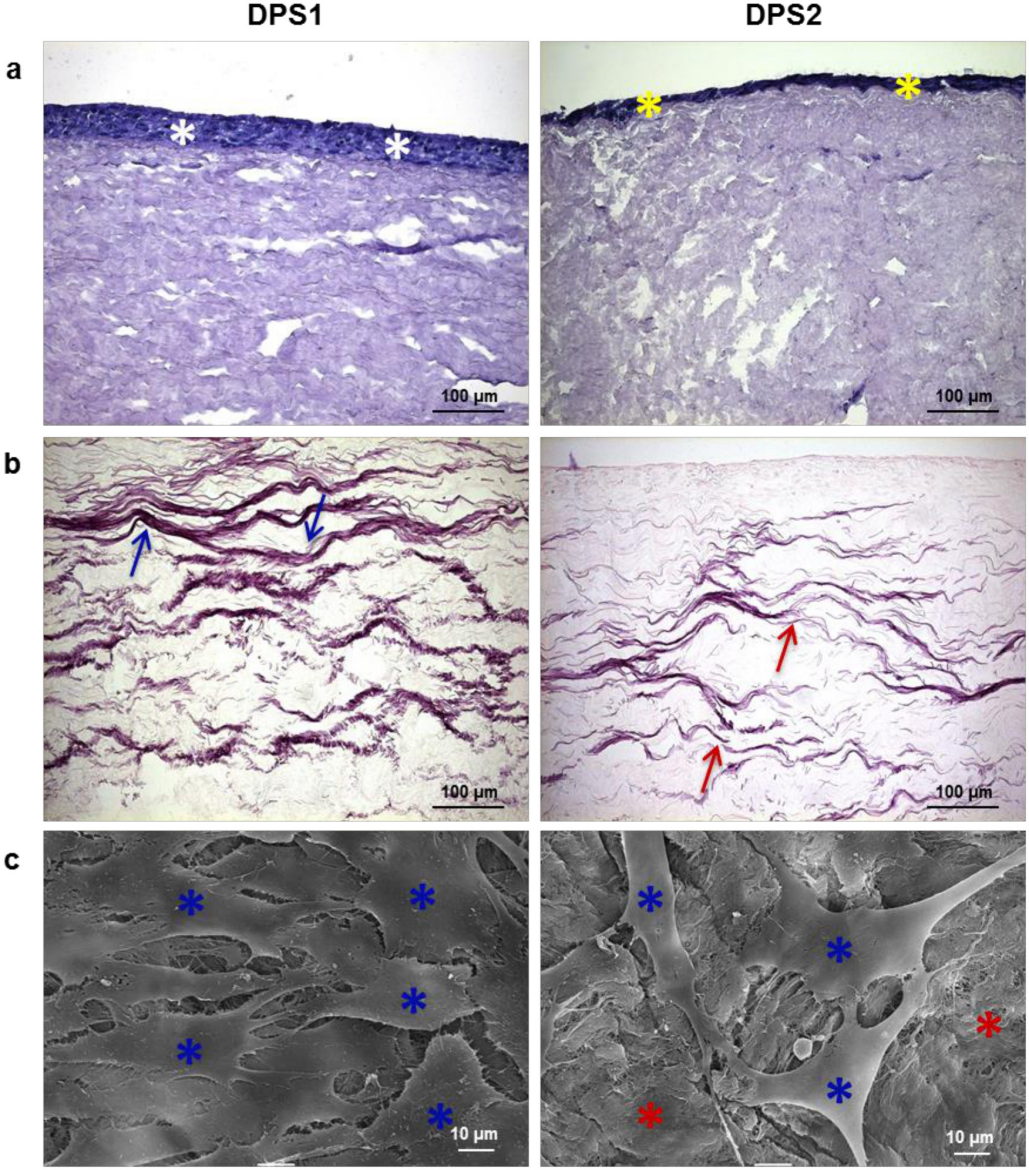
Decellularized bovine pericardium seeded with human fibroblasts. (a) H&E staining at 7 days post-seeding shows that cells are present in the upper layer of both tissues, with a more uniform distribution on DPS1 (white asterisks) with respect to DPS2 (yellow asterisks). (b) The elastin fibers of the sections are clearly defined and continuous in DPS1 (blue arrows), whereas they appear partly distorted and discontinuous in DPS2 (red arrows), as shown by Weigert’s staining. (c) SEM images show that cells (blue asterisks) have colonized the whole surface of DPS1; on the contrary, the DPS2 membrane (red asterisks) is still visible under the cell layer (blue asterisks).

With the aim to identify the protocol that generates the most suitable membrane for the GBR technique, the histological, morphological and the biocompatibility properties of the two acellular bovine pericardia were investigated. As described by Scantlebury [20], one of the design criteria to consider in the development of a GBR membrane is its biocompatibility. The biocompatibility of DPS1 and DPS2 was evaluated by seeding directly human fibroblasts onto these membranes, and maintaining them in culture for 7 days. H&E stained sections of the seeded scaffolds at 7 days reveal that cells grew up to and in contact with both the acellular scaffolds, but showing a better distribution on DPS1 (Fig 10a). In agreement with histological analyses, the results of SEM indicate that human fibroblasts were attached and well distributed on the surface of the pericardium decellularized with protocol P#1; on the contrary, DPS2 was colonized by a smaller number of cells (Fig 10b). These outcomes seem to indicate that decellularization protocol P#1 removed any residual or potentially cytotoxic reagents, thus resulting in a more biocompatible membrane. Apart from being biocompatible, an optimal barrier should be also sufficiently occlusive to avoid fibrous tissue formation [20]. The observation that the seeded fibroblasts do not penetrate the bovine ECM matrix suggests that the decellularized pericardia are able to block soft-tissue ingrowth, allowing the infiltration and activity of bone-forming cells.

The fundamental goal of any decellularization protocol is to remove all cellular material without adversely affecting the composition integrity, mechanical property, and eventual biological activity of the remaining ECM [60]. Apart from collagen, pericardium ECM is composed of a network of elastic fibers, which confers elasticity to the tissue [61,62]. Weigert’s staining of decellularized bovine pericardium samples showed well preserved elastin fibers, indicating the integrity of the ECM, in particular for DPS1 (Fig 10c). Indeed, the elastin fibers maintained wavelike structure in DPS1, whereas they appeared more sparse and partly distorted in DPS2. Based on these observations, the first decellularization method has resulted in a much better preservation of the pericardium ECM integrity compared to the freeze/thaw protocol. The results of the morphological analyses thus suggest that the osmotic shock and detergents treatment did not affect the flexibility of the bovine pericardium. The product obtained with the decellularization protocol P#1 is very promising, since clinical manageability of a GBR membrane is another essential property to consider, in particular in the dental field [63]. A too malleable membrane, or a stiff one, is difficult to use; by contrast, a membrane that maintains a certain degree of flexibility, at least during the insertion phase, is preferred [64,65].

### Evaluation of the host tissue reaction after subcutaneous implantation in rats

**Fig 11:**
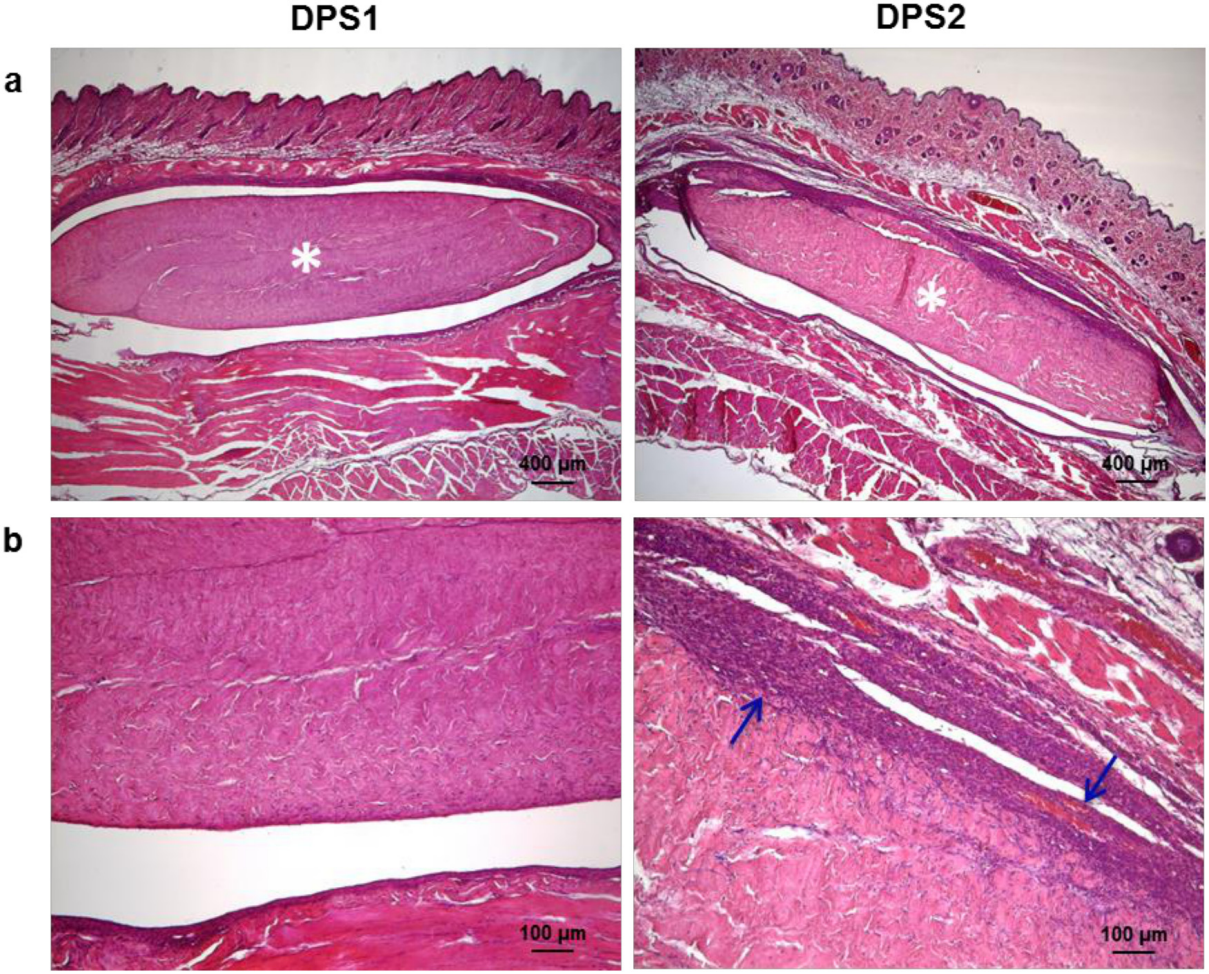
Histological evaluation of host tissue reaction to DPS1 and DPS2 implants. (a) H&E staining performed 7 days after implantation shows that implanted pericardia (white asterisks) are intact, and there is little sign of capsular formation. (b) H&E staining at higher magnification showing a certain degree of inflammation (blue arrows) in DPS2.

Appropriate integration with the surrounding tissue is the ultimate objective of all tissue regeneration techniques, as it is essential that the membrane integrates with the host tissue [63]. A rat subcutaneous implantation model was used to evaluate local tissue reactions following implantation of the two decellularized pericardium membranes intended for GBR. After 7 days from implantation, no signs of rejection were observed for both DPS1 and DPS2 membranes. Nevertheless, different responses of rat tissue to the two decellularized pericardia can be displayed in the histological overviews of Fig 11. In the DPS1 explants, no inflammatory event has been revealed confirming the high tolerability of the material. On the contrary, in the case of DPS2, mononuclear and multinucleated giant cells were found deep into the body of the explants, as well as the appearance of large blood vessels. Based on these observations, it may be speculated that the acellular pericardium scaffold produced with protocol P#1 may be of potential utility for clinical implantation as a GBR membrane.

## Conclusions

The findings of our study are twofold. Driven by the goal of providing an alternative to bone autografts and allografts, we firstly developed a bovine bone graft substitute which closely mimics the natural structure and properties of natural bone ECM. The proposed protocol for bovine bone decellularization consisting in multiple steps of thermal shock, followed by washing with detergent and dehydration with alcohol resulted in a biocompatible, osteoinductive and osteoconductive product. Subsequently, we identified an efficient protocol for decellularizing bovine pericardium. The osmotic shock method seemed superior to the freeze/thaw method for preparing decellularized bovine membranes, as it achieved not only the complete removal of cellular materials but also the preservation of the ECM structure of the bovine pericardium tissue. In addition, the absence of any inflammatory reaction in the host tissue represents an advantage of the method proposed.

In conclusion, we believe that the application of protocols B#3 and P#1 could be considered as a suitable approach to produce decellularized bovine bone and pericardium scaffolds for generating bone substitutes and GBR membranes intended for dental clinical use.

## Supporting Information

S1 Table Human primer sequences. (DOCX)
