# Supplementary material for: Decellularization and Delipidation Protocols of Bovine Bone and Pericardium for Bone Grafting and Guided Bone Regeneration Procedures
Source: PLoS One. 2015 Jul 20;10(7):e0132344. doi: 10.1371/journal.pone.0132344 (PMC4507977; doi:10.1371/journal.pone.0132344)
Supplement: S1 Table — (DOCX) [file pone.0132344.s001.docx]

**S1 Table. Human primer sequences.**

| **gene symbol** | **forward primer (5’→ 3’)** | **reverse primer (5’→ 3’)** | **product length (bp)** |
| --- | --- | --- | --- |
| **ALPL**^a^ | GGCTTCTTCTTGCTGGTGGA | CAAATGTGAAGACGTGGGAATGG | 181 |
| **COL1A1**^b^ | TGAGCCAGCAGATCGAGA | ACCAGTCTCCATGTTGCAGA | 178 |
| **GAPDH**^c^ | TCAACAGCGACACCCAC | GGGTCTCTCTCTTCCTCTTGTG | 203 |
| **IBSP**^d^ | CGAGCCTATGAAGATGAGTACAGC | TGCACCTTCCTGAGTTGAACTTC | 163 |
| **OC**^e^ | GCAGCGAGGTAGTGAAGAGAC | AGCAGAGCGACACCCTA | 193 |
| **ON**^f^ | TGCATGTGTCTTAGTCTTAGTCACC | GCTAACTTAGTGCTTACAGGAACCA | 183 |
| **OPN**^g^ | TGGAAAGCGAGGAGTTGAATGG | GCTCATTGCTCTCATCATTGGC | 192 |
| **OSX**^h^ | TCAGAATCTCAGTTGATAGGGTTTCTC | GGGTACATTCCAGTCCTTCTCC | 183 |
| **RUNX2**^i^ | AGCCTTACCAAACAACACAACAG | CCATATGTCCTCTCAGCTCAGC | 175 |

^a^ALPL, alkaline phosphatase, liver/bone/kidney; ^b^COL1A1, collagen, type I, alpha 1; ^c^GAPDH, glyceraldehyde-3-phosphate dehydrogenase; ^d^IBSP, integrin-binding sialoprotein; ^e^OC, osteocalcin; ^f^ON, osteonectin; ^g^OPN, osteopontin; ^h^OSX, osterix; ^i^RUNX2, runt-related transcription factor 2.
